# Carcinoma Ex Pleomorphic Adenoma of the Parotid Gland: A Rare Case

**DOI:** 10.7759/cureus.25357

**Published:** 2022-05-26

**Authors:** Zaryab Umar, Usman Ilyas, Mohsen S Alshamam, Allison Foster, Rubal Bhangal, Nazaakat Ahmed, Zarwa Idrees

**Affiliations:** 1 Internal Medicine, Icahn School of Medicine at Mount Sinai, Queens Hospital Center, New York, USA; 2 Internal Medicine, NYC Health + Hospitals, New York, USA; 3 Internal Medicine, Icahn School of Medicine at Mount Sinai, New York, USA; 4 Internal Medicine, Icahn School of Medicine at Mount Sinai/NYC Health + Hospitals, New York, USA; 5 Internal Medicine, Queens Hospital Center, New York, USA

**Keywords:** carcinoma ex pleomorphic adenoma, squamous cell carcinoma (scc), squamous cell carcinoma, brain metastasis, malignant pleural effusion, parotid tumor

## Abstract

Cancer is a major cause of morbidity and mortality worldwide, with squamous cell carcinoma (SCC) being the most common type. Even though SCC is the major type of cancer found in the head and neck region, the salivary glands contribute to about 1/20 cases, of which 1/10 are said to be carcinoma ex pleomorphic adenoma (CXPA) type, and the parotid gland is found to be the most common origin of such cases. Although it usually arises later in life, it can grow rapidly, with local symptoms being late findings, if any. Even though fine needle aspiration cytology has low sensitivity for diagnosing such cancer, multiple/repeated biopsies can increase the yield and the accuracy of the test. Surgical resection is the main choice for treatment with postoperative radiation for select cases. Our case presented with CXPA with distant metastasis to multiple sites.

## Introduction

Primary salivary gland malignancies (SGMs) account for only 5% of all head and neck cancers, with the World Health Organization (WHO) describing 11 benign and 22 malignant subtypes of salivary gland tumors [1]. Carcinoma ex pleomorphic adenoma (CXPA) constitutes about 12% of all SGMs, with the majority of cases arising from the parotid gland (67%) [2]. Histopathologically, poorly differentiated adenocarcinoma and undifferentiated carcinoma are the most frequently encountered malignant components of CXPA with squamous cell carcinoma (SCC) being a rare finding [3,4]. Metastatic SGMs are an uncommon finding with only 20% of parotid gland malignancies showing metastatic potential [1]. According to Ali et al., lungs account for 49%, bones for 40%, and the brain for 7% of the total metastasis reported in a study. The study also suggested a distant recurrence percentage of 20% for CXPA [5]. “Occasional case reports exist of adenoid cystic carcinoma metastasizing to the pleural fluid, as well as a rare case of benign metastasizing pleomorphic adenoma, a case of mucoepidermoid carcinoma involving peritoneal and pericardial fluid,” as well as a case of salivary ductal carcinoma in the pleural fluid [6]. Here, we present a case of CXPA with SCC as the malignant component with metastasis to the pleura, lung, brain, liver, and adrenal glands, which, to the best of our knowledge, has never been reported before.

## Case presentation

**Figure 1:**
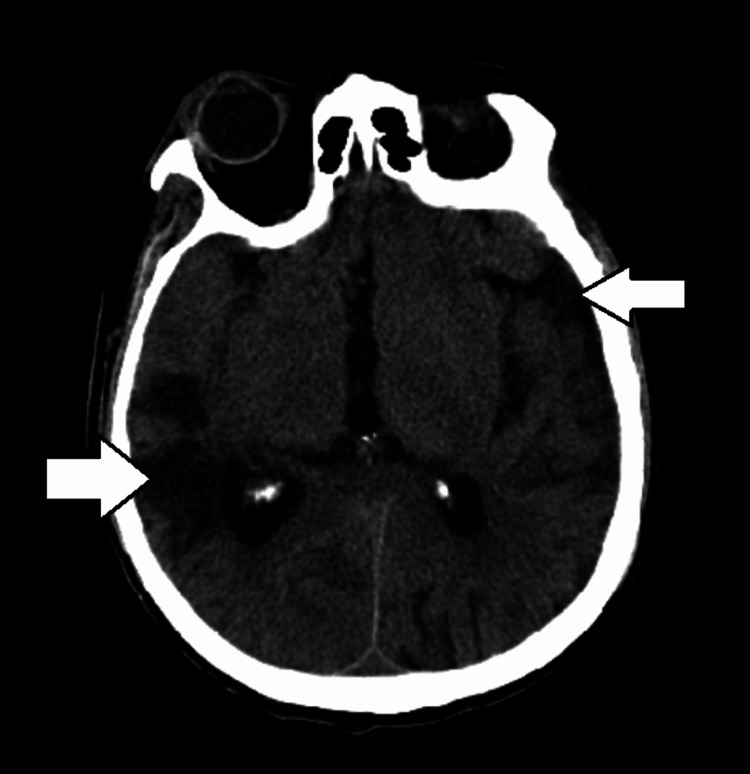
CT scan of the head without contrast showing diffuse encephalomalacia (white arrows).

**Figure 2:**
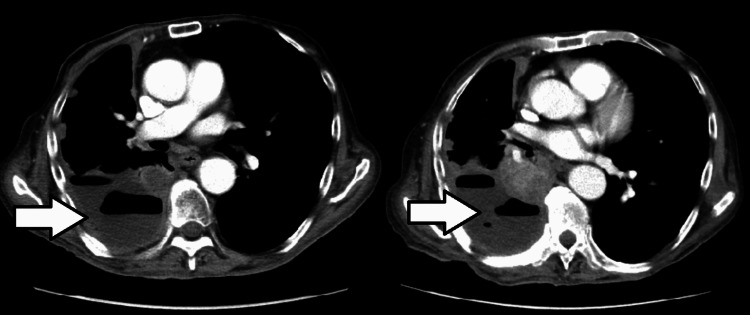
CT scan of the chest (left) showing pleural effusion (white arrow), unchanged from the prior CT scan of the chest (right).

**Figure 3:**
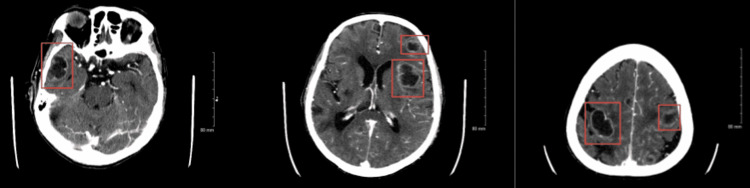
CT scan of the head with contrast showing multiple ring-enhancing lesions suggestive of neoplasm/malignancy.

A 66-year-old former smoker with a past medical history of left parotid gland cancer status post (s/p) radical parotidectomy, temporal bone resection, and nodal dissection with pleural metastasis and recurrent pleural effusions s/p PleurX catheter (BD, Franklin Lakes, NJ) was brought in by the emergency medical services (EMS) post cardiac arrest and admitted to the intensive care unit (ICU). Examination revealed an extremely frail man in a comatose state, with occasional myoclonic jerks and seizures. Computed tomography (CT) scan of the brain without contrast showed diffuse/multifocal encephalomalacia (Figure 1), but a CT scan of the chest without contrast showed no significant changes as compared to the prior CT scans (Figure 2). Electroencephalography (EEG) exhibited a burst suppression pattern suggesting anoxic brain injury. However, a repeat CT scan of the brain without contrast three days later revealed multiple cystic ring-enhancing lesions in the cerebrum and left cerebellum (Figure 3).

**Figure 4:**
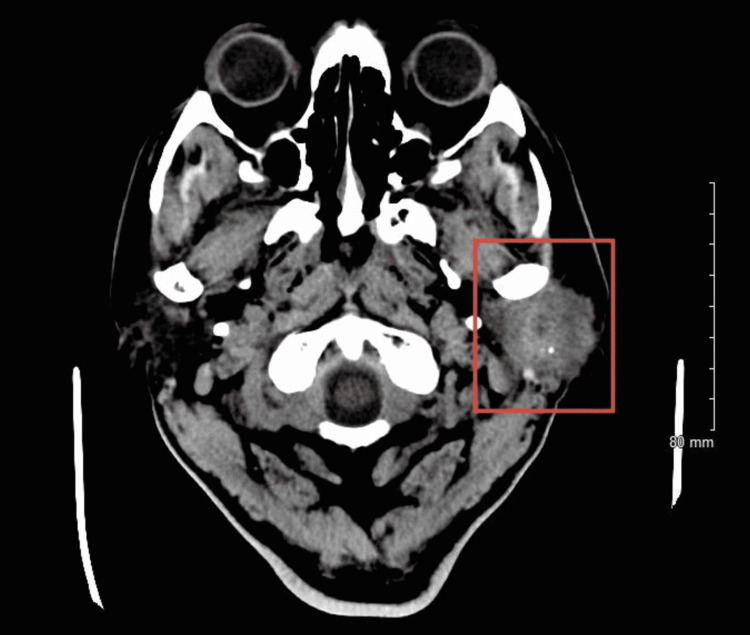
CT soft tissue neck with contrast showing salivary gland tumor centered within the left parotid gland with stranding of the surrounding subcutaneous tissues including the preauricular area as well as inflammatory changes extending toward the cartilaginous segment of the left external auditory canal with associated narrowing.

**Figure 5:**
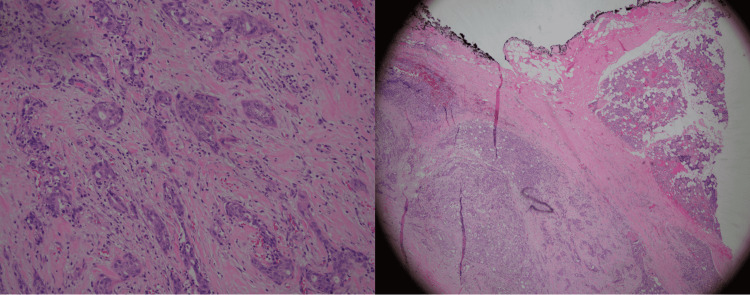
High and low power magnification images of parotid gland resection specimen showing an invasive carcinoma ex pleomorphic adenoma, with squamous cell carcinoma and high-grade adenocarcinoma components.

**Figure 6:**
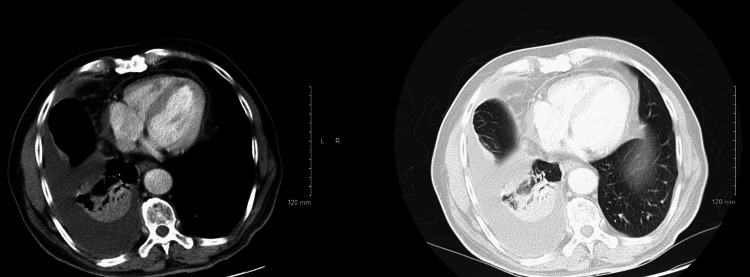
CT chest with contrast evident for right pleural effusion and basilar atelectasis and/or consolidation.

**Figure 7:**
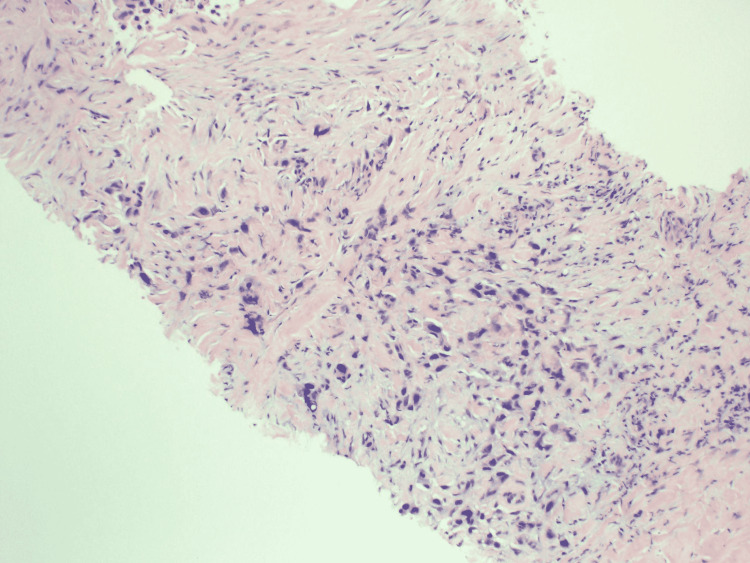
Pleural biopsy showing poorly differentiated epithelial cells infiltrate fibrotic pleural tissue. Tumor cells are positive for p40 and BER-EP4. Findings are consistent with squamous cell carcinoma.

**Figure 8:**
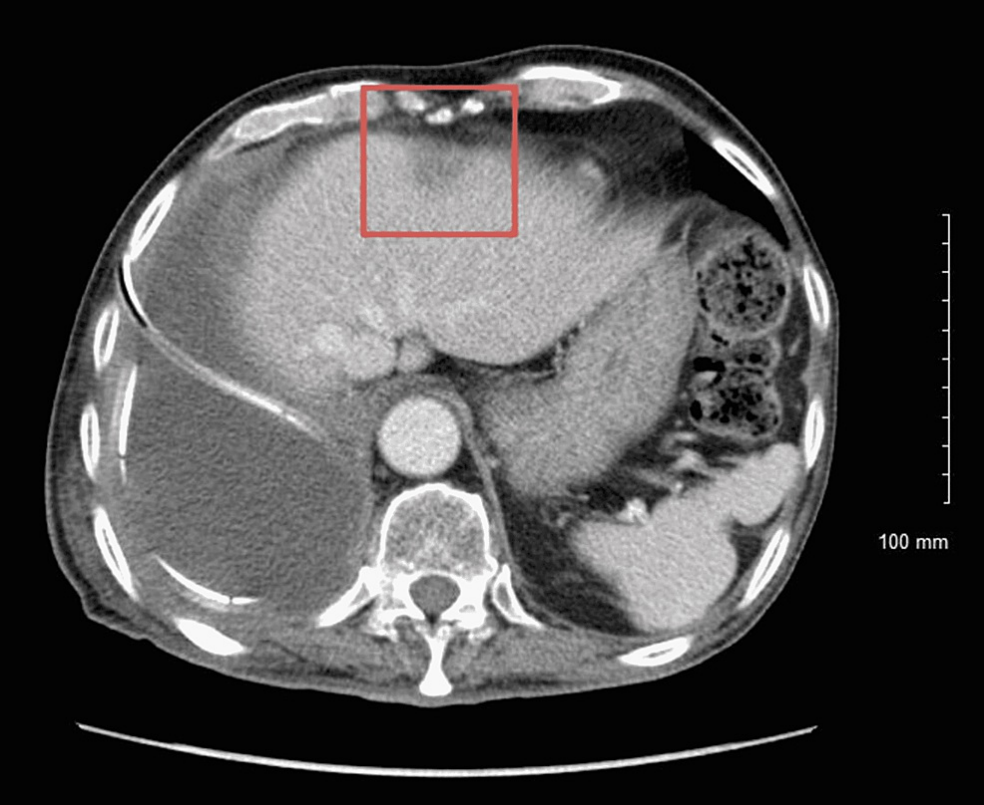
CT scan of the abdomen with contrast showing a 1.6 cm hypodense lesion in the left lobe of the liver.

**Figure 9:**
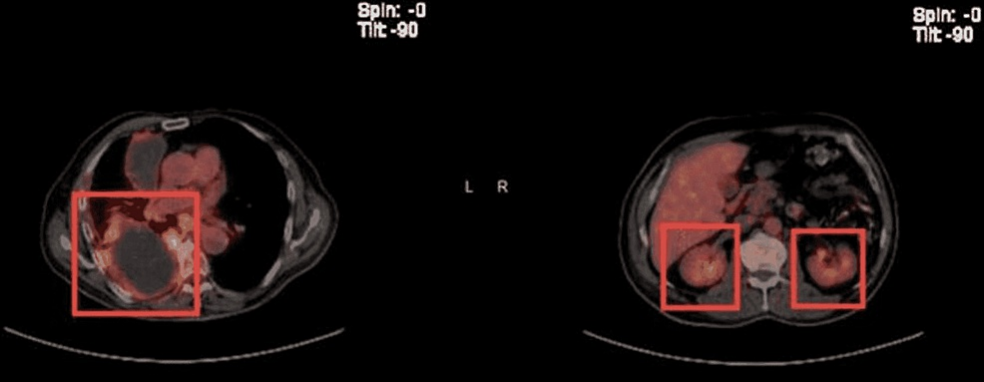
Positron emission tomography scan showing metabolically active foci in localizing to right lower lobe atelectasis and bilateral adrenal glands indicating neoplasm/malignancy.

Before this hospital admission, the patient had undergone a workup revealing left parotid gland cancer, CXPA, with imaging and biopsies shown below (Figures 4, 5). Along his course of illness, he was found to have a collapsed right lung with complete opacification of the right hemithorax consistent with pleural effusion (Figures 6, 7), necessitating further workup to evaluate the cause of the effusion. Thoracocentesis was performed and 1600 milliliters of bloody fluid was drained. Cytology of the pleural fluid showed malignant cells with large and multiple nuclei and a high nuclear-cytoplasmic (N/C) ratio with amphophilic/glassy cytoplasm and scattered mitosis. Cells were compared to the fine needle aspiration sample from the left parotid gland, which shared similar morphology. Immunohistochemical staining was completed (Table 1). A pleural biopsy was done showing poorly differentiated cancer, likely SCC, and likely metastatic in nature (Figure 8). For tumor markers from pleural biopsy, please refer to Table 1. Further workup included a CT scan of the abdomen with contrast showing a 1.6 cm hypodense lesion seen in the left lobe of the liver. A positron emission tomography (PET) showed metabolically active foci in localizing to right lower lobe atelectasis and bilateral adrenal glands, indicating neoplasm/malignancy (Figure 9).

**Table 1:** Pleural fluid cytology and biopsy markers. EMA: epithelial membrane antigen; CK 5/6: cytokeratin 5/6; CK7: cytokeratin 7; TTF-1: thyroid transcription factor 1; PSA: prostate-specific antigen; WT-1: Wilms' tumor 1.

Specimen source	Tumor markers found	Tumor markers absent
Pleural fluid cytology	EMA, CK 5/6, CK7	Calretinin, TTF-1, PSA
Pleural biopsy	AE1/AE2	Calretinin, S-100, TTF-1, WT-1

## Discussion

SCC, although very rare in the parotid gland, is the predominant histological type of head and neck neoplasm [7,8]. SCC usually occurs as a result of metastasis to intraparotid or periparotid lymph nodes from a head and neck cutaneous malignancy, making exclusion of cutaneous malignancy crucial for diagnosis and management [9]. SCC originating in the parotid gland can be either a primary malignancy or arise within a pre-existing pleomorphic adenoma [8]. The latter, known as CXPA, is a rare and aggressive malignancy, defined as an epithelial malignancy arising from primary or recurrent pleomorphic adenoma [10]. The entity comprises approximately 3.6% of all salivary gland tumors, 11.6% of all salivary malignancies, and 6.2% of all pleomorphic adenomas [10]. It has a prevalence rate of 5.6 cases/100,000 malignant tumors and an incidence rate of CXPA is 0.17 tumors/1 million persons [10]. Although it usually presents with an uneventful rapidly growing mass, less commonly, it can present with pain, facial nerve palsy, enlarged lymph nodes, skin fixation, and ulceration [11]. CXPA usually spreads through a direct local extension and metastasis via lymphatic spread to cervical lymph nodes [12]. Distant metastases have been reported to occur in as many as 44% of patients with CXPA [13]. Previously reported sites of hematogenous metastases include the lungs (the most common site), pleura, pharynx, kidney, ocular choroid, liver, bone, brain, and spinal cord [12].

Fine needle aspiration cytology (FNAC) is the preferred initial investigation in tumors of major salivary glands with postoperative pathology assessment being the gold standard for making the diagnosis [10]. FNAC sensitivity is low with multiple biopsies needed to differentiate benign from malignant neoplasm [14]. The most common histological type of CXPA epithelial malignancy is adenocarcinoma (not otherwise specified) but others such as mucoepidermoid carcinoma, SCC, adenosquamous cell carcinoma, adenoid cystic carcinoma, epithelial myoepithelial carcinoma, sarcomatoid carcinoma, acinic cell carcinoma, clear cell carcinoma, and myoepithelial carcinoma are also reported [15]. The term non-invasive carcinoma ex pleomorphic adenoma and the concept of malignant tumor arising in mixed tumors were first introduced by Livolsi and Perzik in 1977 [16]. The proportion of benign versus malignant components can be quite variable [14]. Occasionally, extensive sampling is necessary to find the benign component, and in rare cases, a benign remnant might not be found [14]. In our case as well, only the carcinomatous component was present in the recurrent metastatic lesion. Moreover, the use of 18F-fluorodeoxyglucose (FDG) positron emission tomography (PET) in evaluating salivary gland neoplasms remains unclear [12]. Kim et al. demonstrated an association between high FDG uptake and Glut-1 overexpression in CXPA, which could be potentially clinically applied to differentiate between CXPA and pleomorphic adenoma [12,17], and also showed that in both the initial staging and restaging, FDG PET had a significant impact on the management of patients with salivary malignant tumors [12]. In our case, despite the proper therapeutic management, the patient relapsed one year later. Moreover, FDG PET was useful for the detection of metastases.

Management of CXPA often involves an ablative surgical procedure, which may or may not be followed by reconstructive surgery and radiotherapy [14]. For intracapsular or minimally invasive CXPA localized to the superficial lobe of the parotid gland, superficial parotidectomy is preferred [10]. Whereas for frankly invasive CXPA, total or radical parotidectomy is indicated [10]. Total parotidectomy involves the resection of both the deep and superficial lobes of the parotid, with preservation of the facial nerve if spared by the tumor [10]. However, if neoplasm infiltrates the facial nerve, en bloc resection of the facial nerve, as well as superficial and deep lobes of the parotid gland, is done [10]. If cervical lymph nodes are involved, a functional, modified, or radical dissection is done as well [14]. Postoperative radiotherapy is used when there is suspicion of inadequate resection of the tumor, for high-grade disease, and when there is an invasion of perineural tissues and lymph nodes [10]. Prognostically, tumor size and grade are significant factors in the more widely invasive CXPAs. The extent of tumor infiltration beyond the capsule and, therefore, extraparotid invasion with advanced T stage and lymph node involvement have been found to correlate with CXPA recurrence and survival and they are some of the most reliable prognostic factors. The prognosis also depends on the completeness of the tumor resection, the presence of local recurrence, or distant metastases [10,18]. The prognosis after detection of any cancer progression or recurrence is poor, with a median survival of less than one year [15]. In our case, both regional lymphatic and distant hematogenous spread were identified.

## Conclusions

In summary, recurrence or distant metastases in CXPA are associated with poor prognosis and very low survival. Although distant metastases are rare, our case report emphasizes the importance of long-term follow-up for recurrence as well as local and distant metastases in patients who undergo treatment for CXPA. The case report also aims to shed light on the different histopathological subtypes of SGMs, diagnostic approaches, and management strategies.
